# Changes in corneal thickness following combined cataract and vitreous surgery

**DOI:** 10.1186/s13104-015-1676-9

**Published:** 2015-11-14

**Authors:** Akira Watanabe, Tomohiro Shibata, Hirotsugu Takashina, Hiroshi Tsuneoka

**Affiliations:** Department of Ophthalmology, The Jikei University School of Medicine, 3-25-8 Nishi-shinbashi, Minato-ku, Tokyo, 105-8461 Japan

**Keywords:** Corneal thickness, Combined cataract and vitreous surgery, Vitrectomy, Surgical stress, Micro-incisional vitrectomy system, Epiretinal membrane, Rhegmatogenous retinal detachment

## Abstract

**Background:**

We investigated changes in corneal thickness following combined cataract and vitreous surgery and determined whether such changes could be used as a criterion for evaluating the invasiveness of combined surgery.

**Methods:**

This retrospective, consecutive, comparative study examined 35 eyes that had undergone combined cataract and 23-gauge vitrectomy for epiretinal membrane (ERM) (18 eyes) and rhegmatogenous retinal detachment (RRD) (17 eyes). Corneal thickness was measured before, 1 day, 1 week, and 1 and 3 months after the surgery. Measurements were performed at the center and at points 3 mm superior, inferior, nasal and temporal to the center of the cornea.

**Results:**

In both groups, postoperative corneal thickness at all points showed a significant increase at 1 day after the surgery when compared with the preoperative measurements (p < 0.05, paired *t* test). At the center and inferior points, the mean corneal thickness in the RRD group was thicker than the mean of the ERM group at 1 day after surgery. With the exception of the inferior point, the postoperative data for both groups showed a significant increase in the corneal thickness at 1 week after surgery compared with the preoperative measurements. At 1 week after surgery, the mean corneal thickness in the RRD group at the center, inferior and temporal points was thicker than the mean of the ERM group.

**Conclusions:**

Corneal thickness measurements are useful for assessing the extent of surgical stress that follows combined cataract and vitreous surgery.

**Electronic supplementary material:**

The online version of this article (doi:10.1186/s13104-015-1676-9) contains supplementary material, which is available to authorized users.

## Background

Surgical invasions associated with cataract surgery lead to transient increases in the corneal thickness prior to a gradual recovery to preoperative levels [1–4]. Thus, corneal thickness changes are considered to be useful indicators of corneal endothelial function [2]. In particular, these changes have been used to evaluate the degree of invasion caused by cataract surgery, a procedure that involves the anterior chamber [1].

We have previously reported finding a significant increase in the corneal thickness after vitrectomy, with the thickness subsequently recovering to the preoperative levels at 1 month after the procedure [5]. Additionally, the degree of the corneal thickness increase was affected by the degree of the vitrectomy invasiveness. Thus, it has been shown that corneal thickness measurements can be useful for assessing the extent of the surgical stress that occurs following vitrectomy.

In recent years, utilization of combined cataract surgery and vitrectomy has been increasing due to the spread of the micro-incisional vitrectomy system and microincision cataract surgery approaches. However, there have been no reports that have specifically investigated corneal thickness changes that occur following the use of combined cataract and vitreous surgery.

In this study we compared changes in the corneal thickness following combined cataract surgery and vitrectomy for epiretinal membrane (ERM) and rhegmatogenous retinal detachment (RRD) and determined whether such changes could be used as a criterion for evaluating the invasiveness of combined surgery.

## Methods

This study was designed as a retrospective, consecutive, and comparative study. All study protocols were approved by the Ethics Committee of Jikei University School of Medicine and complied with the Declaration of Helsinki. Informed consent was obtained from all subjects. Our study examined 35 eyes in 35 patients who had undergone combined cataract surgery and 23-gauge transconjunctival vitrectomy for ERM (18 eyes) and RRD (17 eyes) at Jikei University Hospital between January 2009 and July 2010 and for whom the corneal thickness changes could be monitored for at least 3 months after the initial surgery.

The mean ages of the patients were 52.9 ± 12.1 years in the RRD group and 72.0 ± 6.8 years in the ERM group (Student’s *t*-test, p < 0.001). Eyes were graded for nuclear sclerosis of the cataract according to the Emery-Little classification guidelines. In the RRD group, 1 eye was classified as grade 1, 8 eyes as grade 2, and 2 eyes as grade 3, with no eyes classified as grade 4. In the ERM group, 1 eye was classified as grade 1, 11 eyes as grade 2, and 6 eyes as grade 3, with no eyes classified as grade 4. The mean axial length in the RRD group was 25.0 ± 1.71 mm while it was 24.1 ± 1.81 mm in the ERM group (Student’s *t*-test, p = 0.18). The mean of the corneal thickness at the center in the RRD group was 568.2 ± 27.0 µm while it was 554.4 ± 31.9 µm in the ERM group (Student’s *t*-test, p = 0.24). The mean surgical times in RRD and the ERM groups were 104.7 ± 26.2 min and 54.3 ± 10.9 min, respectively (Student’s *t*-test, p < 0.001).

### Cataract and vitreous surgery procedure

All cataract and vitreous surgeries were performed by an experienced surgeon (W.A.) at Jikei University Hospital. In both groups, the cataract surgery was performed via a 2.4-mm superior corneoscleral incision at the 11 o’clock position, with an intraocular lens implanted from same size corneoscleral incision.

We wrote about the common procedures about vitreous surgery in both groups below. A DORC One-Step 23-Gauge Vitrectomy System^®^ was used to insert three ports in the transconjunctiva obliquely to the sclera to perform vitreous surgery. Subsequently, a floating lens and irrigating hand-held lens system were utilized to observe the fundus. If posterior vitreous detachment was not present following the core vitrectomy, it was created during the vitreous surgery. To ensure better visibility of the vitreous, we used triamcinolone acetonide during the vitrectomy.

The subsequent surgical procedures that were used in the two groups were as follows. In the RRD group, all procedures included peripheral vitrectomy with scleral depression around the entire circumference, fluid gas (20 % SF6) exchange, and the performance of internal drainage and laser photocoagulation around the tears during the surgery. After each of the procedures, patients laid in a face-down position for 1–5 days. In the ERM group, we performed core vitrectomy, peripheral vitrectomy with scleral depression around the three ports and ERM peeling during the surgeries. In the ERM group, 9 eyes (50 %) underwent internal limiting membrane (ILM) peeling. ILM removal was performed by flushing 0.125 % ICG solution on the surface of the retina, which made the ILM more visible.

Corneal thickness was measured using a Pentacam^®^ anterior segment analyzer (Oculus, Wetzlar, Germany) before and at 1 day, 1 week, 1 and 3 months after surgery. Measurements were performed at the center and at the points 3 mm superior, inferior, nasal, and temporal to the center.

The degree of anterior segment inflammation was graded before surgery and at 1 day, 1 week, 1 and 3 months after the surgery. The degree of anterior segment inflammation was graded qualitatively by slit-lamp examination using a method adapted from Barraquer et al. [5]. Slit-lamp biomicroscopy was used to evaluate the anterior cell count and conducted in a standardized fashion. Parameters used included dim room illumination, use of the highest voltage lamp, a 0.5 × 2-mm aperture placed in the center area of the pupil, use of a 30° illumination angle, magnification × 16x, and an observation time of 15 s. A clinical inflammation score of the anterior chamber cell count was assessed in each patient as follows: grade 0 = no cells, grade 0.5 = 1–5 cells, grade 1 = 6–15 cells, grade 2 = 16–25 cells, grade 3 = 26–50 cells, Grade 5 = >50 cells [6–8].

Measurement of the corneal endothelial cell count using a specular microscope was performed before surgery and at 3 months after surgery.

We excluded all cases in which there were cataract surgery complications, corneal epithelium removal during the vitrectomy, postoperative corneal epithelium damage, high intraocular pressure >22 mmHg, or in which there had been intraocular surgery performed within 1 year prior to the vitreous surgery.

For 4 weeks after the surgery, all patients were prescribed eye drops containing 0.5 % levofloxacin hydrate, 0.1 % dexamethasone sodium phosphate and 0.1 % bromfenac sodium hydrate. This was followed by the use of eye drops containing 0.5 % levofloxacin hydrate, 0.1 % fluorometholone and 0.1 % bromfenac sodium hydrate for an additional 3 months after the surgery.

### Statistical analysis

All statistical analyses were performed using statistical software programmed by Hisae Yanai (Statcel-3, OMS Publication, Saitama, Japan).

Paired t-test was used to compare the postoperative corneal thickness with the preoperative measurements in ERM and RRD groups.

The independent samples t-test was used in comparisons of ERM and RRD groups.

Wilcoxon signed-rank test was used to compare the postoperative degree of the anterior segment inflammation with the preoperative measurements. Mann–Whitney U test was used in comparisons of ERM and RRD groups.

Two-tailed P values of less than 0.05 were considered to indicate statistical significance.

Two-tailed P values of less than 0.005 (Bonferroni adjustments) were considered to indicate statistical significance for multiple comparison.

## Results

**Fig. 1:**
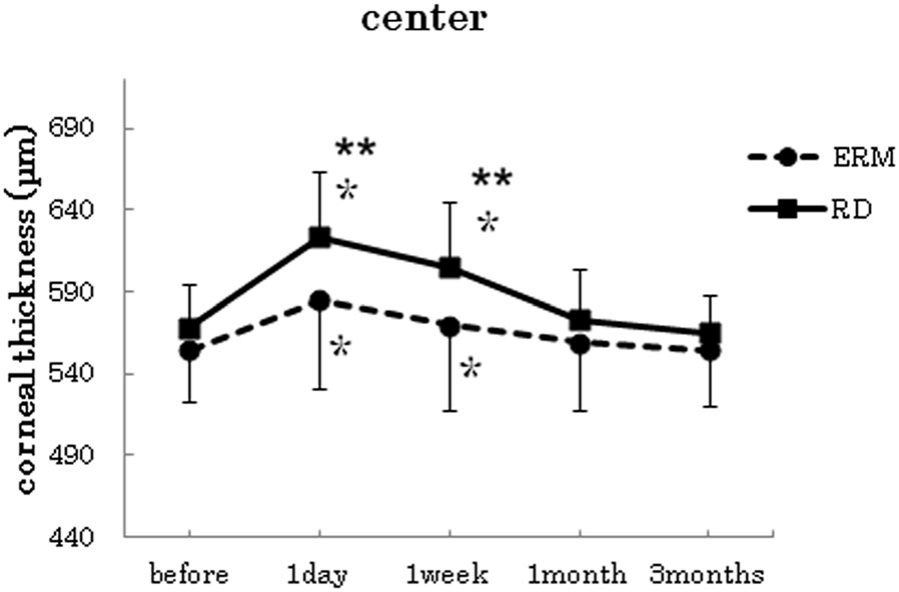
Changes in corneal thickness at the center. In both groups, the postoperative corneal thickness was significantly increased at 1 day and 1 week after surgery when compared to the preoperative measurements [*p < 0.005 (Bonferroni adjustments), paired t-test]. The mean corneal thickness at 1 day and 1 week after surgery in the RRD group was thicker than the mean of the ERM group (**p < 0.05, *t*-test)

**Fig. 2:**
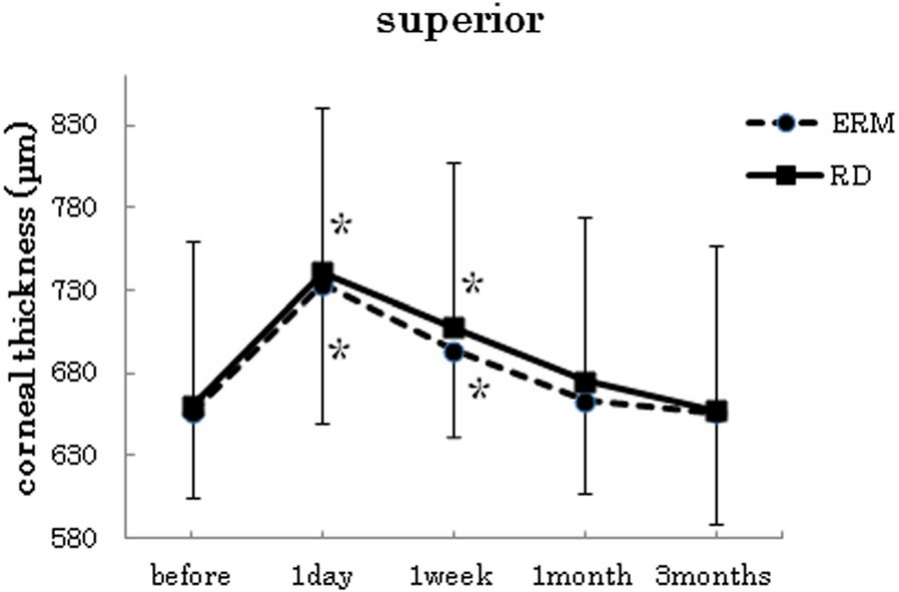
Changes in corneal thickness at the points 3 mm superior. In both groups, the postoperative corneal thickness was significantly increased at 1 day and 1 week after surgery when compared to the preoperative measurements (p < 0.005, paired t-test). There was no difference in the proportion of the change of the corneal thickness in both the ERM and RRD groups (p < 0.05, *t*-test)

**Fig. 3:**
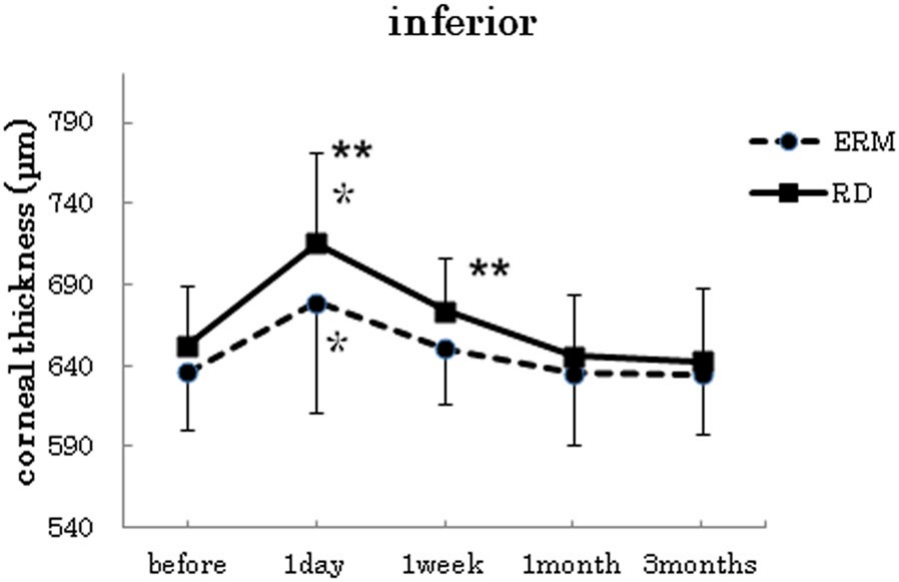
Changes in corneal thickness at the points 3 mm inferior. In both groups, the postoperative corneal thickness was significantly increased at 1 day after surgery when compared to the preoperative measurements (p < 0.005, paired t-test). The mean corneal thickness at 1 day and 1 week after surgery in the RRD group was thicker than the mean of the ERM group (p < 0.05, *t*-test)

**Fig. 4:**
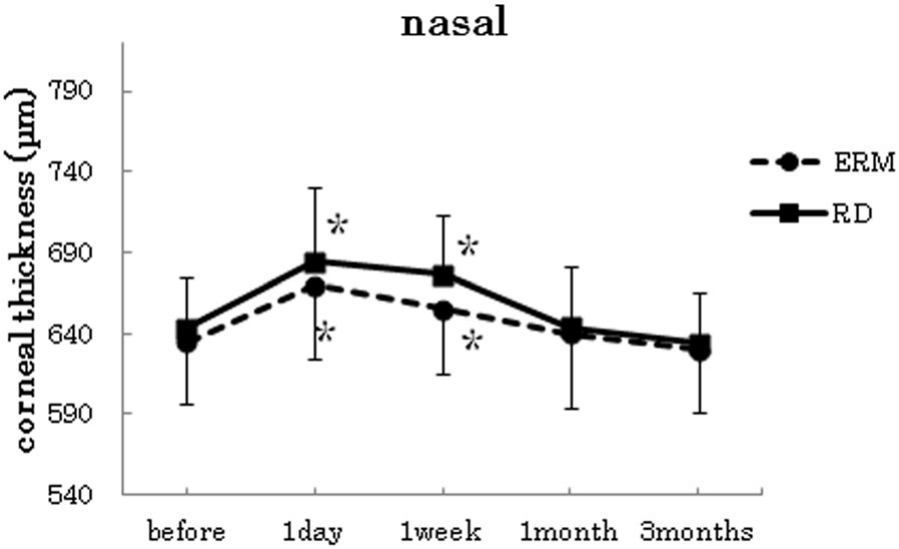
Changes in corneal thickness at the points 3 mm nasal. In both groups, the postoperative corneal thickness was significantly increased at 1 day and 1 week after surgery when compared to the preoperative measurements (p < 0.005, paired t-test)

**Fig. 5:**
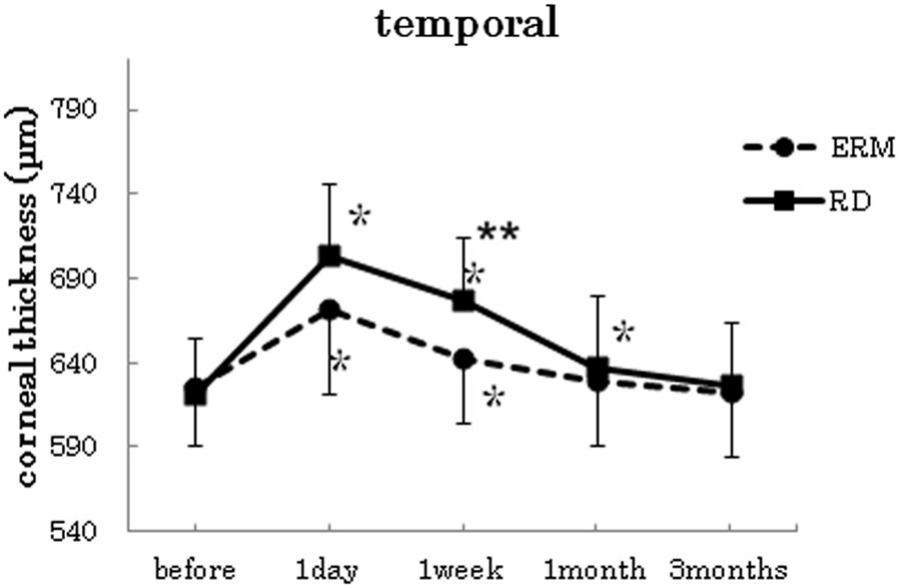
Changes in corneal thickness at the points 3 mm temporal. In both groups, the postoperative corneal thickness was significantly increased at 1 day and 1 week after surgery when compared to the preoperative measurements (p < 0.005, paired t-test). In the RRD group, a significant increase in the corneal thickness was noted for the mean of the postoperative data at 1 month after the surgery when compared with the preoperative measurements (p < 0.005, paired t-test). The mean corneal thickness at 1 week after surgery in the RRD group was thicker than the mean of the ERM group (p < 0.05, *t*-test)

Figures 1, 2, 3, 4, 5 show the results for the comparisons of the corneal thickness changes between the ERM and RRD groups. No significant differences were apparent for the preoperative thickness measurements at any of the measurement points between the two groups (*t*-test center: p = 0.18, superior: p = 0.81, inferior: p = 0.30, nasal: p = 0.51, temporal: p = 0.69).

In both groups, the postoperative corneal thickness was significantly increased at 1 day after surgery when compared to the preoperative measurements (paired t-test at all points : p < 0 0.001). At the center and inferior points, the mean corneal thickness at 1 day after surgery in the RRD group was thicker than the mean of the ERM group. (*t*-test center: p = 0.018, superior: p = 0.77, inferior: p = 0.027, nasal: p = 0.41, temporal: p = 0.14).

With the exception of the inferior point (paired t-test ERM p = 0.17, RRD p = 0.07), the postoperative data for both groups showed a significant increase in the corneal thickness at 1 week after surgery when compared with the preoperative measurements (paired t-test at all points : p < 0.001). At the center, inferior and temporal points, the mean corneal thickness at 1 week after surgery in the RRD group was thicker than the mean observed in the ERM group (*t*-test center: p = 0.007, superior: p = 0.52, inferior: p = 0.04, nasal: p = 0.515, temporal: p = 0.017).

In the ERM group, no significant increases in the corneal thickness were observed for the mean of postoperative data at 1 month after surgery when compared to the preoperative measurements (paired *t*-test center: p = 0.27, superior: p = 0.36, inferior: p = 0.97, nasal: p = 0.33, temporal: p = 0.50). In the RRD group, no significant increases in the corneal thickness were observed for the mean of postoperative data at 1 month after surgery when compared to the preoperative measurements (paired *t*-test center: p = 0.29, superior: p = 0.06, inferior: p = 0.52, nasal: p = 0.92, temporal: p = 0.008). In both groups, however, there were no significant increases in the corneal thickness for the mean of the postoperative data at 3 months after the surgery when compared with preoperative measurements (ERM; paired *t*-test center: p = 0.96, superior: p = 0.99, inferior: p = 0.91, nasal: p = 0.31, temporal: p = 0.56), (RRD; paired *t*-test center: p = 0.54, superior: p = 0.60, inferior: p = 0.34, nasal: p = 0.23, temporal: p = 0.42).

Additional file 1: Table S1 summarize the degree of the anterior segment inflammation before surgery and at 1 day, 1 week, 1 month, and 3 months after the surgery. In both groups, the postoperative anterior segment inflammation score showed a significant increase for 1 month after the surgery when compared with the preoperative anterior segment inflammation score (Wilcoxon signed-rank test, ERM; 1 day: p < 0.001, 1 week: p < 0.001, 1 month : p = 0.002, RRD; 1 day: p < 0.001, 1 week : p < 0.001, 1 month : p < 0.001*)*. No significant differences were apparent in the anterior segment inflammation score for both groups at 3 months after the surgery when compared with preoperative anterior segment inflammation score (Wilcoxon signed-rank test, ERM: p = 0.11, RRD: p = 0.21).

In the RRD group, the scores of the anterior segment inflammation before surgery (p = 0.014) and at 1 day (p < 0.001), 1 week (p < 0.001), 1 month (p = 0.007), and 3 months after surgery (p = 0.026) were all higher than the scores in the ERM group. (Mann–Whitney U test, *p* < 0.05).

In the RRD group, the mean corneal endothelial cell count was 2696 ± 319 preoperatively and 2534 ± 300 at 3 months after surgery. In the ERM group, the mean corneal endothelial cell count was 2547 ± 318 preoperatively and 2445 ± 299 at 3 months after surgery. In both groups, no significant differences were apparent in the mean corneal endothelial cell count at 3 months after the surgery (paired t-test; ERM: p = 0.56, RRD: p = 0.62).

## Discussion

Corneal thickness has been used as invasion indicators for cataract surgery. It has been shown to be useful for evaluating the degree of corneal endothelial dysfunction due to anterior chamber procedures during cataract surgery or postoperative intraocular inflammation [1–5]. Moreover, the extent of the corneal thickness increase after cataract surgery has been reported to be correlated with future corneal endothelial disorders [2]. Thus, the use of corneal thickness as an indicator for evaluating surgical invasiveness may be of great clinical significance.

In a previous study, we found a significant increase in the corneal thickness after vitrectomy only [5]. By 1 month, however, these increases had disappeared and the thickness had recovered to the original preoperative levels, similar to that seen for cataract surgery. In addition, we also noted that the degree of the increase in the corneal thickness was affected by the degree of invasiveness of the vitrectomy. Therefore, corneal thickness measurements may be useful for assessing the extent of the surgical stress for example longer operating time, scleral depression, fluid gas exchange and laser photocoagulation that occurs following vitrectomy.

In this study, we compared the corneal thickness changes following cataract and vitreous combined surgery in both the RRD and ERM groups. In the ERM group, cataract surgery, core vitrectomy, peripheral vitrectomy with scleral depression around the three ports, and ERM peeling were all performed. On the other hand, in the RRD group, cataract surgery, core vitrectomy, peripheral vitrectomy with scleral depression around the entire circumference, internal drainage, fluid-gas exchange and laser photocoagulation around tears were performed during surgery. After the surgery, patients maintained a face-down position for 1–5 days. Fluid-gas exchange has been previously reported to increase the inflammation in the anterior chamber [9]. In the RRD group, besides surgery itself, postsurgical treatment of RRD by retinal tamponade with intraocular gas in a face-down position and air-fluid exchange that pushes the iris-lens diaphragm forward are also considered invasive with respect to the eye. This may affect corneal thickness. When the process and the duration of the surgery are taken into consideration, it would be expected that the invasiveness of the vitreous surgery and treatment for RRD should be higher in the RRD group than in the ERM group. In addition to these findings in the RRD group, the score of the anterior segment inflammation before surgery and at 1 day, 1 week, 1 month, and 3 months after the surgery were found to be higher than the scores in the ERM group. At the center of the cornea, the postoperative corneal thickness at all points showed a significant increase by 1 week after surgery when compared with the preoperative measurements in both groups. In addition, the proportion of the corneal thickness increase in the RRD group was higher than that observed in the ERM group. These findings suggest that corneal thickness measurements may be useful for assessing the extent of the surgical stress for that occurs following combined cataract and vitreous surgery.

At points 3 mm superior to the center, there was no difference in the proportion of the change of the corneal thickness in both the ERM and RRD groups. During cataract surgery, corneal thickness changes were used as the evaluation criteria for surgical stress. The proportion of the change in the corneal thickness was the largest around the wound that occurred after the cataract surgery [10]. Based on these results, we suspected that the cataract surgery wound had the greatest influence on the corneal thickness changes at points that were 3 mm superior to the center. Therefore, in addition to examinations of the whole volume of the cornea, corneal thickness changes at the focal points of the cornea also need to be taken into consideration when evaluating the invasion of surgery.

Although our study determined that the degree of the corneal thickness change differed between the temporal and nasal points, the reason for this remains unclear. It has speculated that other procedures may also be affecting the changes in the corneal thickness. In addition, other factors are known to affect to the change of the corneal thickness in cataract and vitreous combined surgery. These factors include corneal endothelial dysfunction due to anterior chamber procedures during cataract surgery or postoperative intraocular inflammation, corneal or corneoscleral incision, and the condition of the corneal epithelium and ocular pressure.

Increased corneal thickness is known to increase measured values of intraocular pressure (IOP) [11–13]. Values for IOP measured after vitreous surgery may thus be higher than the actual value. However, the extent to which increased corneal thickness due to transient corneal endothelial injury by surgical invasion actually affects measured IOP values remains unknown.

Given the retrospective nature of the present study, future prospective studies will need to be undertaken. Also, since the number of cases examined in this study was small, future studies must be rigorously consistent with respect to both the underlying diseases and the surgical procedures.

## Conclusions

Corneal thickness measurements are useful for assessing the extent of surgical stress that follows combined cataract and vitreous surgery.

## Additional file

10.1186/s13104-015-1676-9 Degree of the anterior segment inflammation score before and after surgery.

## References

[1] Suzuki H, Takahashi H, Hori J, Hiraoka M, Igarashi T, Shiwa T (2006). Phacoemulsification associated corneal damage evaluated by corneal volume. Am J Ophthalmol.

[2] Lundberg B, Jonsson M, Behndig A (2005). Postoperative corneal swelling correlates strongly to corneal endothelial cell loss after phacoemulsification cataract surgery. Am J Ophthalmol.

[3] Behndig A, Lundberg B (2002). Transient corneal edema after phacoemulsification: comparison of 3 viscoelastic regimens. J Cataract Refract Surg.

[4] Nissen JN, Ehlers N, Frost-Larsen K, Sørensen T (1993). The effect of topical steroid on postoperative corneal edema and endothelial cell loss after intracapsular cataract extraction. Acta Ophthalmol. (Copenh).

[5] Watanabe A, Shibata T, Takashina H, Ogawa S, Tsuneoka H (2012). Changes in corneal thickness following vitreous surgery. Clin Ophthalmol..

[6] Jabs DA, Nussenblatt RB, Rosenbaum JT, Standardization of Uveitis Nomenclature (SUN) Working Group (2005). Standardization of uveitis nomenclature for reporting clinical data. Results of the First International Workshop. Am J Ophthalmol.

[7] Barraquer RI, Alvarez de Toledo JP, Montané D, Escoto RM, Garcia Torres C, Bennani-Tazzi M (1998). Fixed-dose combination of 0.1 % diclofenac plus 0.3 % tobramycin ophthalmic solution for inflammation after cataract surgery: a randomized, comparative, active treatment-controlled trial. Eur J Ophthalmol.

[8] Rizzo S, Genovesi-Ebert F, Murri S, Belting C, Vento A, Cresti F (2006). 25-gauge, sutureless vitrectomy and standard 20-gauge pars plana vitrectomy in idiopathic epiretinal membrane surgery: a comparative pilot study. Graefes Arch Clin Exp Ophthalmol.

[9] Constable IJ, Swann DA (1975). Vitreous substitution with air. Arch Ophthalmol.

[10] Li YJ, Kim HJ, Joo CK (2011). Early changes in corneal edema following torsional phacoemulsification using anterior segment optical coherence tomography and Scheimpflug photography. Jpn J Ophthalmol.

[11] Georgalas I, Pagoulatos D, Koutsandrea C, Papaconstantinou D (2013). Don’t forget that central corneal thickness affects intraocular pressure. BMJ.

[12] Hagerb A, Dave H, Wiegand W (2005). Corneal pachymetry and intraocular pressure. Klin Monbl Augenheilkd..

[13] Emara B, Probst LE, Tingey DP, Kennedy DW, Willms LJ, Machat J (1998). Correlation of intraocular pressure and central corneal thickness in normal myopic eyes and after laser in situ keratomileusis. J Cataract Refract Surg.

