# Supplementary material for: Changes in corneal thickness following combined cataract and vitreous surgery
Source: BMC Res Notes. 2015 Nov 14;8:674. doi: 10.1186/s13104-015-1676-9 (PMC4644329; doi:10.1186/s13104-015-1676-9)
Supplement: Supplementary file 1 — 10.1186/s13104-015-1676-9 Degree of the anterior segment inflammation score before and after surgery. [file 13104_2015_1676_MOESM1_ESM.docx]

Table S1 Degree of the anterior segment inflammation score before and after surgery

|  | **ERM** | **RRD** | **p** |
| --- | --- | --- | --- |
| before | 0±0 | 0.15±0.019 | 0.020** |
| 1day | 1.56±0.51* | 3.12±0.33* | <0.001** |
| 1week | 0.72±0.26* | 1.53±0.72* | <0.001** |
| 1M | 0.42±0.31* | 0.71±0.25* | <0.001** |
| 3M | 0.08±0.19 | 0.26±0.26 | 0.043** |

before=before vitrectomy; 1day = 1 day after vitrectomy; 1 week=1 week after vitrectomy;

1 month=1 month after vitrectomy; 3 months=3 months after vitrectomy.

* : A significant increase compared with preoperative measurement (Wilcoxon signed-rank test, *p* < 0.005 (Bonferroni adjustments))

** : The mean anterior segment inflammation score in the RRD group was higher than the mean observed in the ERM group (Mann–Whitney U test, *p* < 0.05)
